# Tackling the challenge of controlling the spin with electric field

**DOI:** 10.1093/nsr/nwaa267

**Published:** 2020-12-04

**Authors:** Roberta Sessoli

**Affiliations:** Dipartimento di Chimica ‘Ugo Schiff’ & INSTM RU, Università degli Studi di Firenze, Italy

**Figure 1.**
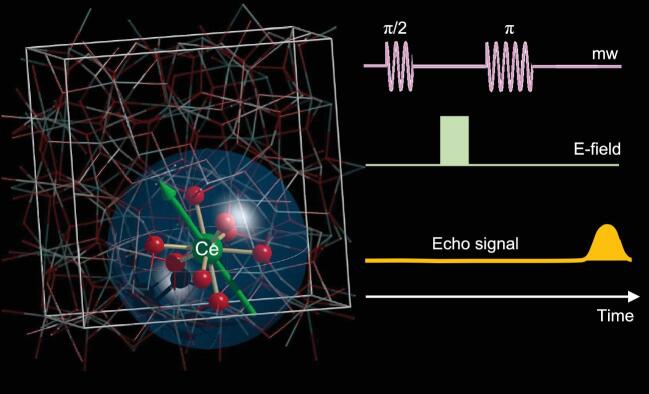
Diluted Ce^3+^ ions (f^1^, *J *= 5/2) substituting diamagnetic Y^3+^ ions in the YAG lattice are operated as spin qubits using mw pulses, as commonly done in pulsed electron paramagnetic resonance spectroscopy (color code: Ce, green; Y, cyan; Al, grey; O, red). Liu Z *et al.* [4] have added an electric field pulse and demonstrated that it acts as a phase gate modifying the spin echo signal. The large spin-orbit coupling of Ce^3+^ ion is a key ingredient for coherent electric field control of the spin, while the rigid garnet lattice warrants long spin coherence also at moderate temperatures.

Electric (*E*) fields are commonly employed to control magnetization in some classes of materials such as multiferroics. However, the extreme confinement of the electric field, for instance under the tip of a scanning tunnel microscope, makes it an ideal tool to manipulate individual spins, generating interest in spins as potential quantum bits. Spins are in fact intrinsic quantum systems that can be manipulated easily by means of pulses of electromagnetic microwaves, as commonly done in magnetic resonance spectroscopies. Although the coupling usually involves the magnetic field of microwaves, the first experiments with *E-*field pulses date back to the 1970s when Mims investigated the symmetry of the lanthanide ion coordination environment [1]. A linear effect is characteristic of an acentric environment. Renewed interest in spin-electric coupling is motivated by recent advances in scanning probe spectroscopies able to perform single atom CW [2] and pulsed [3] EPR spectroscopy. These exceptional tools make possible the optimization of spin-electric coupling in the qubit and exploration of the potential for gate control. In this respect, ensemble measurements (diluted single crystals or frozen solutions) provide an immense playground. Excluding rare exceptions, spin-electric coupling is enhanced by spin-orbit coupling. Lanthanide-based materials with the orbitally unquenched contribution of 4f electrons are promising candidates. Liu Z *et al.* recently coordinated a pulsed EPR study on Ce^3+^ ions (f^1^, *S *= 1/2 and *J *= 5/2 but with a well isolated ground doublet) diluted in a polar diamagnetic crystal of yttrium aluminum garnet (YAG), as shown in Fig. 1 [4]. The standard  π/2 − *t* − π Hahn sequence generating an echo was modified by insertion of an *E-*field pulse. The Hahn echo intensity was found to depend on both *E-*pulse intensity and length, demonstrating that the *E-*field is controlling the spin system. The effect, often quantified as a frequency shift per V m^−1^, is about one order of magnitude larger than that detected in molecular qubits based on 3d ions. In the same study, the authors used the *E-*pulses to control evolution of the spin system through a phase gate and to implement the Deutsch–Jozsa algorithm, a well-known example of a deterministic quantum algorithm which is exponentially faster than its classical analogue.

However, an *E-*driven phase gate is usable only if a large number of operations can be made before the coherence is lost. Efficient operation requires strong spin-electric coupling but the same mechanisms that originate the coupling (spin-orbit interaction and atomic displacements) are also responsible for magnetic relaxation and loss of coherence. The Ce^3+^ in YAG has good coherence lifetime, 15 μs at 10 K, thanks to its rigid lattice and low nuclear spin concentration. Interestingly, a contemporaneous investigation by Liu J *et al.* highlighted even stronger spin-electric coupling in a holmium(III) polyoxometalate [5]. The strength of the effect has been rationalized considering the coupling with a vibration mode of the Ho coordination sphere. Shorter coherence lifetimes were observed, although these were enhanced when protected at clock transitions.

In conclusion, recent advances in *E-*field coherent control of spin qubits suggest that single spin qubit operation is viable and particularly promising for discrete molecular systems. However, optimization of coherence time and spin-electric coupling poses antithetic conditions and remains an open challenge for chemists and physicists working in this fascinating field.

***Conflict of interest statement*.** None declared.
